# Assessment tool for hospital admissions related to medications: development and validation in older patients

**DOI:** 10.1007/s11096-018-0768-8

**Published:** 2018-12-26

**Authors:** Thomas G. H. Kempen, Mariann Hedström, Hanna Olsson, Amanda Johansson, Sara Ottosson, Yousif Al-Sammak, Ulrika Gillespie

**Affiliations:** 1 Hospital Pharmacy Department, Uppsala University Hospital, Uppsala, Sweden; 2 Department of Medical Sciences, Uppsala University, Uppsala, Sweden; 3 Department of Pharmaceutical Biosciences, Uppsala University, Uppsala, Sweden; 4 Department of Public Health and Caring Sciences, Uppsala University, Uppsala, Sweden

**Keywords:** Assessment tool, Drug-related problems, Elderly, Hospital admissions, Medication-related admissions, Sweden

## Abstract

*Background* Medication-related hospital admissions (MRAs) are frequently used to measure outcomes in studies involving medication reviews. The process of identifying MRAs is subjective and time-consuming, and practical, validated alternatives are required. *Objective* The aim of this study was to develop and validate a practical tool to identify MRAs. *Setting* Uppsala University Hospital, Sweden. *Method* We reviewed existing literature on methods to identify MRAs. The tool AT-HARM10 was developed using an iterative process including content validity and feasibility testing. The tool’s inter-rater reliability (IRR) and criterion-related validity (CRV) were assessed: four pairs of either final-year undergraduate or postgraduate pharmacy students applied the tool to one of two batches of 50 older patients’ hospital admissions. Assessment of the same 100 admissions by two experienced clinicians acted as gold standard. *Main outcome measure* Cohen’s and Fleiss’ kappa for IRR, and sensitivity, specificity, and positive and negative predictive value for CRV. *Results* AT-HARM10 consists of ten closed questions to distinguish between admissions that are unlikely to be and those that are possibly medication-related. The IRR was moderate to substantial (Cohen’s kappa values were 0.45–0.75 and Fleiss’ kappa values were 0.46 and 0.58). The sensitivity and specificity values were 70/86% and 74/70%, positive and negative predictive values were 73/74% and 71/83% respectively. Both AT-HARM10 and the gold standard identified approximately 50% of the admissions as MRAs. *Conclusion* AT-HARM10 has been developed as a practical tool to identify MRAs and the tool is valid for use in older patients by final-year undergraduate and postgraduate pharmacy students.

**Electronic supplementary material:**

The online version of this article (10.1007/s11096-018-0768-8) contains supplementary material, which is available to authorized users.

## Impacts on practice

- The tool, ATHARM10, can be used to identify medication-related hospital admissions in older patients as a valid outcome in clinical research.
- The ATHARM10 tool potentially decreases research costs as it can be used by final-year undergraduate or postgraduate pharmacy students with little involvement of clinical experts.

## Introduction

Medication-related problems (MRPs) are highly prevalent among older patients taking multiple medications, and can lead to a negative impact on health outcomes and increasing healthcare costs [1]. MRPs are defined here as “undesirable patient experiences that involve medication therapy and that actually or potentially interfere with desired patient outcomes” [2]. These not only involve adverse drug reactions (ADRs) to prescribed medication, but can also involve problems such as inappropriate prescribing and non-compliance, and problems related to over-the-counter (OTC) medications. Up to 30% of older patients’ hospital admissions can be attributed to MRPs [3, 4]. Over half of these medication-related admissions (MRAs) are possibly or definitely preventable [5–8]. Healthcare interventions, such as medication reviews, have been introduced to promote appropriate prescribing, increase the correct use of medications and decrease the incidence of MRAs [9–11]. As such interventions primarily target medications and medication-related issues, MRAs are consequently the main admissions that they can impact. The use of the incidence of MRAs as an outcome measure has therefore recently been proposed as part of a standard core-outcome set for studies involving medication reviews in older patients taking multiple medications [12].

However, there is no validated method of identifying MRAs. The most common method is for an expert panel to assess the patient’s medical record and reach a consensus [11, 13, 14]. The use of an expert panel is often viewed as the “gold standard”. In medicine, a gold standard refers to a test, a treatment, or a benchmark that is the best available under reasonable conditions [15]. An expert panel assessment involves the use of senior clinicians or researchers and is often time-consuming, which makes it a costly method. To lower research costs, involvement of under- or postgraduate students should be considered in academic research [16]. However, the identification of MRAs by either an expert panel or students will inevitably introduce a degree of subjectivism. The use of standardized methods that have been tested for reliability and validity can minimize this subjectivism [15, 17, 18]. Validated tools for determining the association between ADRs and hospital admissions, such as the Naranjo algorithm, are often used [19–23]. Unfortunately, none of these tools have been developed to identify the full range of MRPs that can result in hospital admissions, leading to the risk of underestimating the incidence of MRAs. In fact, non-compliance has been found to be one of the largest contributors to MRAs [5, 24, 25]. One standardized method to identify the full range of MRAs in older people has recently been developed [26]. However, the performance of this method in terms of predictive validity, sensitivity and specificity has not yet been validated and it also uses an expert panel for the assessment.

Our research group is currently carrying out a cluster-randomized controlled trial [the Medication Reviews Bridging Healthcare (MedBridge) study] which aims to evaluate the effects of comprehensive medication reviews in hospitalized older patients [27]. One of the outcome measures is the incidence of MRAs, and more than 5000 readmissions are expected during the 12-month follow-up period. There is thus a need for a practical assessment tool, defined as being possible to use by under- or postgraduate pharmacy students, within a limited time frame and without the need for an expert panel.

## Aim of the study

The aim of this study was to develop and validate a practical tool to identify MRAs.

## Ethics approval

This study is part of the MedBridge study, which has received ethical approval from the Swedish Central Ethical Review Board (CEPN Ö21-2016). Additional approval for the medical record screening of non-MedBridge study participants was received from Uppsala University Hospital, in compliance with local regulations.

## Method

### Development of the tool

The first part of the study was an unstructured review of the available literature to find existing methods or tools used to identify MRAs. Relevant articles related to MRAs were obtained using the Medline database in February 2016. Articles in languages other than English or Swedish and abstract-only articles were excluded. The literature search identified several articles using various methods to identify MRAs [3, 4, 6, 11, 13, 25, 28–40]. Some studies included a variety of MRPs [3, 4, 11, 25, 31, 32, 34, 35, 37–39] as causes of MRAs, often referring to existing MRP classification systems like the eight categories by Strand et al. [2]. Some studies only included ADRs [6, 13, 29, 30, 33, 36, 40]. When assessing causality, one study [28] used completely implicit methodology while all the others [3, 4, 6, 11, 13, 26, 29–38] used established criteria such as the Naranjo probability scale [21], the Hallas criteria [22] or the Kramer algorithm [20] to guide an expert panel (consisting of physicians or pharmacists with various degrees of seniority).

The results of the literature search were used to develop the preliminary version of our tool. We defined an MRA as a hospital admission of which an MRP is either the main cause for admission or a significantly contributing cause for admission (i.e. without the MRP, the patient would not have been admitted). Elements from established tools and previously published studies were listed to include all relevant categories of MRPs with the potential to cause, or contribute to, a hospital admission, and to include reasons for classifying admissions as non-medication-related [2, 5, 11, 19–21, 25, 41–46]. Overlapping elements were combined and reformulated. The tool was designed in the form of a questionnaire with yes/no answers, and it consisted of ten questions in the final version (Table 1). Explicit lists with medication-specific triggers or clinical rules were excluded to make the tool less time-consuming. The rationale for choosing the questionnaire format was that it would be easy to use. Questions 1–3 in the tool are used to identify admissions that are *unlikely* to be medication-related (U1–3), while questions 4–10 are used to identify *possibly* MRAs (P4–10). The assessment is finished as soon as the answer is “yes” to any of the questions. Only if all the questions are answered “no”, the assessment is indecisive and should still be examined by an expert panel. The terms *unlikely* and *possibly* are in line with the causality terminology of the UMC-WHO system [23]. The reason for not distinguishing between other degrees of certainty or preventability was to make the assessment not too complex and time-consuming. For the same reason, we decided that only a limited amount of data would be available for the assessments (admission notes, medication list upon admission, laboratory data during hospital stay, and discharge summary). Instructions for use, including examples, were developed for the tool (“Supplementary material”).

**Table 1:** The final version of the assessment tool for identifying hospital admissions related to medications (AT-HARM10)

Question	References	MRP category [2]
U1. Was the admission caused by an infection or a previously undiagnosed disease (e.g. diabetes or heart failure) that is not medication-related?	[44, 45]	n.a.
U2. Was the admission caused by progression of a previously diagnosed disease that is not medication-related?	[19, 21, 41, 46]	n.a.
U3. Was the admission caused by physical trauma, substance intoxication, social circumstances or allergies that are not medication-related?	[19, 21, 41, 46]	n.a.
P4. Is it hinted or stated in the medical record that the admission was medication-related (including non-compliance)?	[21]	Any MRP category
P5. Might (side) effects of the medications the patient was taking (prescribed or not prescribed) prior to hospitalization have caused the admission (including over-treatment)?	[5, 19–21, 25, 41]	5. Overdosage 6. Adverse drug reaction 8. Drug use without indication
P6. Are there abnormal laboratory results or vital signs that could be medication-related and might have caused the admission?	[5, 20, 21, 42]	2. Improper drug selection 5. Overdosage
P7. Was there any drug-drug interaction or drug-disease interaction (i.e. a contraindication) that might have caused the admission?	[11, 25, 43]	2. Improper drug selection 7. Drug interaction
P8. Did the patient have any previously diagnosed untreated or sub-optimally treated (e.g. dose too low) indications that might have caused the admission?	[5, 25, 43]	1. Untreated indication 2. Improper drug selection 3. Subtherapeutic dosage
P9. Was the patient admitted because of a problem with the dosage form or pharmaceutical formulation (i.e. failure to receive the medication)?	[5, 11, 25]	4. Failure to receive drug
P10. Is the cause of the admission a response to cessation or withdrawal of medication therapy?	[47]	6. Adverse drug reaction

Three questions are used to identify admissions that are *unlikely* to be medication-related (U1-U3) and seven questions (P4-P10) to identify *possible* medication-related admissions. References to criteria used in existing tools or former studies that were identified in the literature search, and the corresponding eight medication-related problem (MRP) categories by Strand et al. [2], are listed for each question (1–8)

*n.a.* not applicable, *MRP* medication-related problem

### Content validity

The concept of content validity relies on the assumption that a tool is intrinsically valid if all relevant aspects and no irrelevant aspects are included [17]. The content validity of the preliminary version of the tool was assessed using a questionnaire to score each question for relevance, understandability and completeness. Seven clinical pharmacists were asked to fill in the questionnaire and were encouraged to suggest additions or modifications. The questions that gained low scores were deleted or changed. Two questions (P9–10; Table 1) were added and seven questions were rephrased. The number of questions was eventually set at ten in the final version: Assessment Tool for identifying Hospital Admissions Related to Medications (AT-HARM10).

One hundred admissions of patients aged 65 years or older, discharged from two internal medicine wards at Uppsala University Hospital between January and April 2016, were randomly selected to test the content validity of the final version of the tool. Data were obtained from the hospital’s electronic medical record system (Cosmic^®^). Seven pharmacy students (five final-year undergraduates, one doctoral student and one postgraduate clinical pharmacy student) received information about the tool (“Supplementary material”) and a 1-h training by two researchers (YA-S and UG) on how to use the tool. One of the researchers (UG) was an experienced clinical pharmacist and researcher. The students then applied the tool to five admissions (none of which were subsequently used in the study). The results of the assessments were discussed in plenum. Each student assessed 50 or 100 of these admissions (see “Inter-rater reliability” section), resulting in a total of 400 assessments. The number of the specific question within AT-HARM10, that was used to classify each admission as *unlikely* to be or *possibly* medication-related, was recorded for these 400 assessments, to determine whether all questions were relevant and which questions were used the most.

### Clinical utility

An assessment tool must be practical and it should be possible to use it within a reasonable time frame [48]. Fifteen clinical pharmacists each applied the tool to 10 randomly selected admissions of patients aged 65 years or older, discharged from one internal medicine ward and one geriatric ward at Uppsala University Hospital (Sweden) between December 2015 and January 2016. Data were obtained from the hospital’s electronic medical record system (Cosmic^®^). The pharmacists evaluated whether the limited patient data provided for the assessments were sufficient to satisfactorily answer the questions. They also discussed the tool’s appropriateness and user-friendliness. Time spent on assessing the admissions was measured and averaged to determine the acceptability of the time taken.

### Inter-rater reliability

The inter-rater reliability (IRR) refers to the degree of consistency among the assessors when assessing the same set of samples [15]. The seven participants of the content validity test (five undergraduate and two postgraduate pharmacy students) were divided into four pairs (one student was part of both pair 1 and 4 to enable the formation of four pairs). The pairs consisted of either only postgraduate students or only undergraduate students (Table 2).

**Table 2:** Grouping for the inter-rater reliability test

Pair	Admission number
1	1–50
2^a^	1–50
3	51–100
4	51–100

^a^Postgraduate students

The tool, instructions for use and examples (“Supplementary material”), and anonymized patient data from 50 admissions (either 1–50 or 51–100 of the 100 admissions used for the content validity test, see “Content validity” section) were sent to each assessor. These patient data consisted of copies of the electronic medical records: admission notes, medication list upon admission, laboratory data during hospital stay and discharge summary.

Each assessor then independently applied AT-HARM10 to their assigned 50 hospital admissions, classifying them as either *unlikely* to be or *possibly* MRAs. After assessing the admissions separately, each pair of assessors discussed the admissions that they disagreed on to reach consensus. Cohen’s kappa within each couple and Fleiss’ kappa between pairs assessing the same admissions were calculated to determine the IRR. Cohen’s kappa measures the agreement between two assessors and Fleiss’ kappa is used in cases with more than two assessors [15, 49]. The kappa values were then interpreted according to Hammond et al. [15] (Text Box 1).

**Text Box 1:** Interpretation of kappa values for inferring strength of agreement [15]

Kappa	Strength of agreement
0	None
0–0.2	Slight
0.21–0.4	Fair
0.41–0.6	Moderate
0.61–0.8	Substantial
0.81–1.0	Almost perfect

### Criterion-related validity

The traditional definition of criterion-related validity (CRV) is a measure of the validity of a tool by correlating the results with those from some other measure, ideally a gold standard, which has been used and accepted in the field [17]. The sensitivity, specificity, positive predictive value (PPV) and negative predictive value (NPV), as defined in Text Box 2, were used to measure the CRV.

**Text Box 2:** Criterion-related validity definitions for AT-HARM10 (based on definitions in Hammond et al. [15])

Sensitivity: The probability that the tool will detect *possible* medication-related admissions (MRAs) among the admissions that are truly related to medication according to the gold standard
Specificity: The probability that the tool will detect *unlikely* to be MRAs among admissions that are truly not related to medication according to the gold standard
Positive predictive value (PPV): The percentage of admissions identified by the tool as *possibly* MRAs that are truly related to medication according to the gold standard
Negative predictive value (NPV): The percentage of admissions identified by the tool as *unlikely* to be MRAs that are truly not related to medication according to the gold standard

We defined the gold standard as an expert panel of experienced clinicians. One consultant physician (geriatrics and primary care specialist) and one senior clinical pharmacist and researcher (UG) assessed the same 100 admissions as the four pairs of study assessors, see “Inter-rater reliability” section. The experts had access to all patient data in the electronic medical journal. These data included medical notes from all healthcare professionals, medication histories, and laboratory results from both hospital and primary care facilities. They did not use AT-HARM10 or any other tool for the evaluations. The experts assessed the admissions individually, classifying them as either *unlikely* to be or *possibly* an MRA. They then discussed the cases on which they disagreed to reach consensus and hence created the gold standard for the 100 admissions. In cases where consensus could not be reached, a third expert was available for a decisive vote.

The sensitivity, specificity, PPV and NPV were calculated according to the formula in Table 3. The primary outcomes for the CRV were the consensus results of the AT-HARM10 assessments of all 100 patient admissions, performed by couples 1 + 3 and 2 + 4, compared to the gold standard.

**Table 3:** Formula used to calculate sensitivity, specificity, positive predictive value (PPV) and negative predictive value (NPV) as outcome measures for the criterion-related validity

	Gold standard *unlikely* MRA	Gold standard *possibly* MRA	Total
ATHARM10 *unlikely* MRA	A (true negative)	B (false negative)	A + B
ATHARM10 *possibly* MRA	C (false positive)	D (true positive)	C + D
Total	A + C	B + D	
$$Sensitivity = \frac{D}{D + B};\,Specificity = \frac{A}{A + C};\,PPV = \frac{D}{D + C};\,NPV = \frac{A}{A + B}$$			

*MRA* medication-related admission

## Results

Clarification: all results in this section relate to the testing of the final tool, AT-HARM10, not to the work done on earlier versions of the tool as this was covered in the Methods section above.

### Content validity

**Fig. 1:**
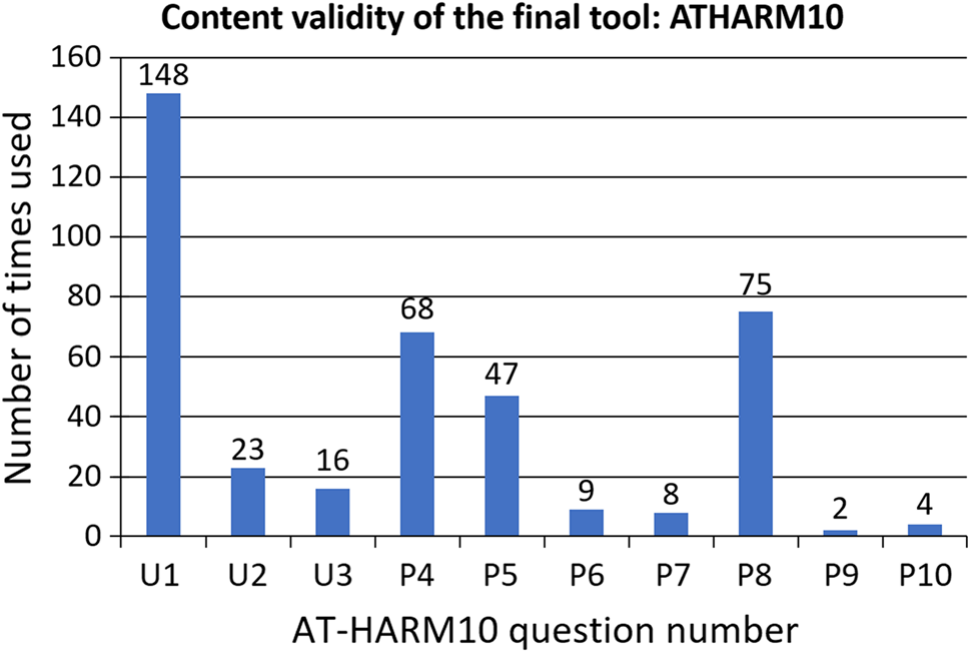
The number of times each of the questions (U1–3 and P4–10) were used by within 400 assessments of in total 100 admissions

After the development phase resulting in the final tool, AT-HARM10, the question that was used most often when assessing the admissions was question U1 (148/400), followed by questions P8 (75/400), P4 (68/400) and P5 (47/400), see Fig. 1. All the questions were used at least twice. In all cases, at least one question was answered with “Yes”, hence no expert panel was needed.

### Clinical utility

According to the fifteen assessing clinical pharmacists, AT-HARM10 was sufficiently relevant and user-friendly and was easy to use; no further changes were suggested. The limited data obtained from the example patients were deemed sufficient to answer the questions in the tool. The time used to assess each admission was on average 5.7 (range 2.5–14) min.

### Inter-rater reliability

The strength of agreement was substantial for one pair of study assessors (Cohen’s kappa 0.75) and moderate for the other three (Cohen’s kappa 0.45–0.57), see Table 4. The strength of agreement among all assessors was moderate (Fleiss’ kappa 0.58 and 0.46 for the two batches of data).

**Table 4:** Inter-rater reliability between assessors using AT-HARM10

Pairs	Admissions assessed	Cohen’s kappa	Fleiss’ kappa
1	1–50	0.75	0.58
2^a^	1–50	0.45	
3	51–100	0.52	0.46
4	51–100	0.57	

^a^Postgraduate students

### Criterion-related validity

The gold standard experts reached consensus for all 100 assessments, which resulted in 50% of the 100 admissions being classified as *unlikely* to be and 50% as *possibly* a MRA. Pairs 1 + 3 and 2 + 4 assigned 52% and 42%, respectively, to *unlikely, and* 48% and 58%, respectively, to *possibly.* The sensitivity was 70% and 86% for pairs 1 + 3 and 2 + 4, respectively. The specificity was 70% and 74% for 1 + 3 and 2 + 4, respectively. The PPV and NPV were 73%/74% for 1 + 3/2 + 4 and 71%/83% for 1 + 3/2 + 4, respectively (Table 5).

**Table 5:** Criterion-related validity of AT-HARM10

	Gold standard		Total pairs	Sens. (%)	Spec. (%)	PPV (%)	NPV (%)
	*Unlikely*	*Possibly*					
Pair 1 + 3							
*Unlikely*	37	15	52 (52%)	70	74	73	71
*Possibly*	13	35	48 (48%)				
Pair 2 + 4							
*Unlikely*	35	7	42 (42%)	86	70	74	83
*Possibly*	15	33	58 (58%)				
Total gold standard	50 (50%)	50 (50%)					

The number of *unlikely* to be and *possibly* medication-related admissions, as classified by the gold standard and the study pairs, are provided

*Sens.* sensitivity, *Spec.* specificity, *PPV* positive predictive value, *NPV* negative predictive value

## Discussion

We have developed a tool for identifying MRAs, AT-HARM10, and validated the tool for use in older patients by final-year undergraduate or postgraduate pharmacy students. The IRR, with Cohen’s kappa values ranging from 0.45 to 0.75 and Fleiss’ kappa values of 0.46 and 0.58, was moderate to substantial, and the CRV, with values ranging from 70 to 86%, was moderate to high [15]. No expert panel was needed for the assessments using AT-HARM10. An assessment took on average 6 (range 2.5–14) min, meaning that the expected 5000 readmissions in the MedBridge study can be assessed in 500 h. The recently published method by Thevelin et al. [26] had similar IRR (Cohen’s kappa values from 0.33 to 0.86 and a Fleiss’ kappa of 0.41), but the mean time needed to assess an admission was considerably longer (23 ± 6 min) and the method involves the use of experts in geriatric medicine.

Approximately half of all admissions in this study were considered *possibly* medication-related, according to both the gold standard experts (50%) and the AT-HARM10 assessments (48–58%). These figures are higher than anything we have identified in the literature (the highest was 30% [3, 4]). There are several possible explanations for this: we included all types of MRPs in our assessments and we did not assess the degree of certainty (i.e. we had the pragmatic view that for some chronic diseases, typically diabetes mellitus and congestive heart failure, it is hard to rule out the possibility that suboptimal therapy had contributed to the admission).

Another way of looking at the results is that, with ATHARM10, researchers can quickly rule out 50% of the admissions as *unlikely* to be medication-related. Hence, should one wish to elaborate on preventability or degree of certainty, for example using an expert panel, only half of the hospital admissions need to be evaluated in more depth.

The tool was developed in close collaboration with the intended future users, which helped to keep the process on track. Also, since AT-HARM10 has been developed and tested over a period of nearly 2 years, we feel that all possible aspects of the tool and its use have been carefully considered. All ten questions were based on criteria from previous studies or existing tools, and the tool takes into consideration all possible MRP categories. A broad range of relevant validation parameters have successfully been tested and all of these are standard parameters in validation studies [15, 17, 48, 49].

One aspect that was deemed important throughout the study was the care taken to thoroughly introduce and demonstrate the use of the tool before starting any assessments. The tool is therefore not just the ten questions, it also includes the whole package of instructions and examples (“Supplementary material”), relying on thorough elaboration and group discussion before starting the assessments.

There are several limitations to this study. First, AT-HARM10 does not assess the degree of certainty or the preventability of MRAs. This was deliberate, as we wanted to keep the tool simple and straightforward, specifically for measuring the incidence of MRAs as a research outcome. Second, the study would perhaps have benefitted from more input from the medical profession; i.e. by including experts in diagnostics. The gold standard may not necessarily be totally reliable; however, an expert panel with full access to the patient data is the best we currently have. Third, we did not perform a comprehensive systematic review of the literature. We may have missed relevant studies in our literature review. However, no validated methods were found in a recent more structured literature review either [26]. We consider AT-HARM10 a valid and practical tool to identify MRAs in older patients and a valuable addition to already existing research methods. It is however, at this moment, unclear if the tool can be used as it is in patients younger than 65 years, by other healthcare students (e.g. medical students) and in other countries. The information sources used for the assessment may need to be adjusted for the local situation. To confirm the results of this study, AT-HARM10 would benefit from further validation performed by independent, national and international research groups with different patient populations.

## Conclusion

AT-HARM10 has been developed as a practical tool to identify MRAs and the tool is valid for use in older patients by final-year undergraduate and postgraduate pharmacy students.

## Electronic supplementary material

Below is the link to the electronic supplementary material.

Supplementary material 1 (DOCX 36 kb)
